# Complete genome sequence of *Rhodococcus qingshengii* phage Perlina

**DOI:** 10.1128/mra.00869-24

**Published:** 2024-10-08

**Authors:** James Jaryenneh, Rohan Krishna, Joseph S. Schoeniger, Catherine M. Mageeney

**Affiliations:** 1 Sandia National Laboratories, Livermore, California, USA; Queens College Department of Biology, Queens, New York, USA

**Keywords:** bacteriophage genetics, *Rhodococcus*

## Abstract

*Rhodococcus* phage Perlina is a novel phage isolated on *Rhodococcus qingshengii* S10. Perlina encodes 112 open reading frames with typical phage structural genes identified and 3 tRNAs (tRNA-Ile, tRNA-Met, and tRNA-Asn). Few close relatives can be identified at the nucleotide level, suggesting a new phage species.

## ANNOUNCEMENT

Bacteriophages continue to be a source of new gene products, information about phage evolution, and tools (1, 2). *Rhodococcus* species are important members of soil microbiomes, important for bioremediation and bioconversion processes for the production of novel compounds (3).

*Rhodococcus* phage Perlina was isolated on *Rhodococcus qingshengii* S10 (S10) from a home compost pile (GPS 37.93132569198107,–121.7123907950711), using the enriched isolation method (4). We added phage buffer to the soil, inverted it, and allowed it to settle before filtering through a 0.2-µm filter. This filtrate was incubated with fresh LB and a 1:100 overnight S10 culture and incubated shaking at 30°C, 220 rpm overnight, filtered, and used for plaque assays. S10 was grown at 30°C in LB broth shaking at 220 RPM overnight for all experiments. Plaques were purified three times and amplified to create a high titer lysate (HTL). DNA was extracted from the HTL using the Norgen Biotek Phage DNA Isolation Kit (default protocol) after PEG precipitation. A DNA library was prepared using the Illumina DNA prep tagmentation kit with Illumina Nextera DNA Unique Dual index adapters (set D).

**Fig 1:**
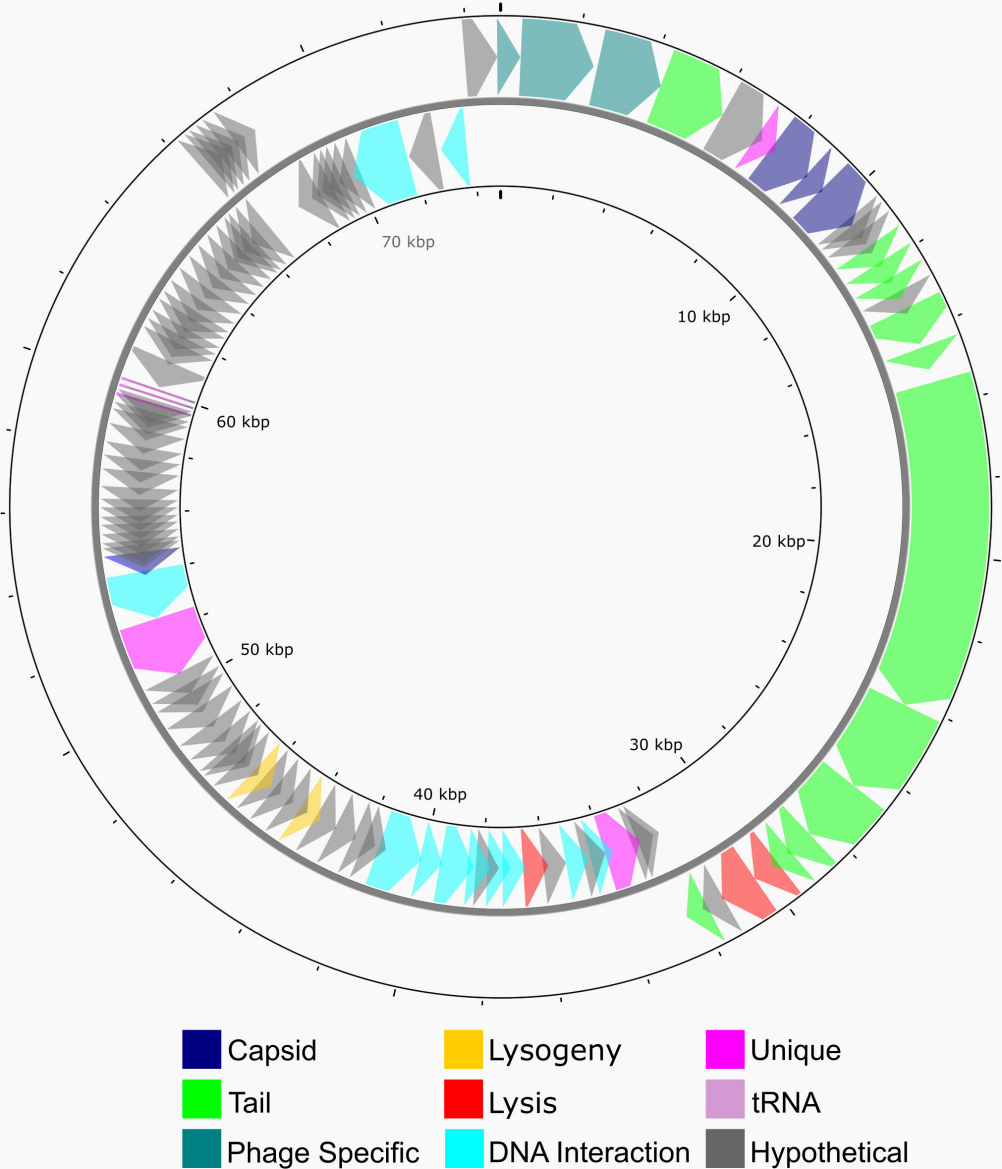
Perlina’s complete genome map. Predicted coding sequences are denoted by colored arrows indicating transcriptional direction and putative function.

Genomic DNA from Perlina was sequenced to 3,499× coverage using the Illumina Nextseq 550 platform (2 × 75 bp). Reads were filtered using BBduk (v36.11; parameters: ktrim = r, k  =  21, mink  =  11, hdist  =  1) (http://jgi.doe.gov/data-and-tools/bb-tools/), reducing 14,991,251 raw reads to 14,991,059 high-quality reads. The resultant reads were assembled using SpAdes (v3.9.0)(5). The 74,872 bp genome is circularly permuted, as determined by mapping the raw reads across the genome and not observing any regions with above-average coverage, with a G + C content of 54.2% (Fig. 1). Positional annotation was completed using MultiPhate(6) (v0.5) with Glimmer(7) (v3.02), Prodigal(8) (v2.6.3), and Phanotate(9) (v0.13.0) turned on and web-based GeneMarkS (10) to call 112 open reading frames (ORFs). Functional annotation was completed using web-based BLASTp (11) and HHpred (12) searching against Pfam-A_v35 (13), PDB_mmCIF70 (14), and TIGERFams_v15.0 (15) databases. Putative functions could be assigned to 38 ORFs (33.9%), most of which were phage structural proteins. The tail assembly chaperone protein was predicted to be translated using a −2 translational frameshift (16). In addition, tRNAscan-SE(17) (v1.3.1) was used to identify three tRNAs specifying methionine, isoleucine, and aspartate. Default parameters were used unless otherwise noted.

Perlina has few close relatives at the nucleotide level; using BLAST (11), the closest phage identified was *Rhodococcus* phage ReqiDocB7(18)(NC_023706.1), with 77.36% identity over 11% of the genome. Both terminase genes have amino acid (AA) homology (small subunit 60.14% AA-ID over 100% of the protein; large subunit 76.76% AA- ID over 95% of the protein) with ReqiDocB7 suggesting that Perlina uses a headful packaging strategy (18). Perlina is taxonomically characterized as Viruses; Duplodnaviria; Heunggongvirae; Uroviricota; Caudoviricetes; and unclassified Caudoviricetes, based on the NCBI taxonomy server (19). Perlina encodes a few interesting genes that warrant follow-up: (i) cas4 endonuclease, (ii) split lysin A gene, (iii) auxiliary metabolic gene, CobT-like cobalamin biosynthesis protein; and (iv) Mu-like com protein.

## Data Availability

The Perlina genome sequence is available at GenBank accession number PP782351. Raw reads were deposited at NCBI Sequence Read Archive BioProject accession number PRJNA1134717.
